# Perspectives from Young South African and Zimbabwean Women on Attributes of Four (Placebo) Vaginal Microbicide Delivery Forms

**DOI:** 10.1007/s10461-019-02576-8

**Published:** 2019-06-28

**Authors:** R. Weinrib, E. N. Browne, M. K. Shapley-Quinn, A. van der Straten, M. Beksinska, N. Mgodi, P. Musara, N. Mphili, J. L. Schwartz, S. Ju, H. Hanif, E. T. Montgomery, on behalf of the Quatro Study Team

**Affiliations:** 1 Women’s Global Health Imperative, RTI International, San Francisco, CA USA; 2 Department of Medicine, Center for AIDS Prevention Studies, University of San Francisco, San Francisco, CA USA; 3 MatCH Research Unit, Department of Obstetrics and Gynaecology, School of Clinical Sciences, University of the Witwatersrand, Durban, South Africa; 4 University of Zimbabwe College of Health Sciences-Clinical Trials Research Centre, Harare, Zimbabwe; 5 Eastern Virginia Medical School, CONRAD, Arlington, VA USA; 6 RTI Health Solutions, RTI International, Barcelona, Spain

**Keywords:** Acceptability, End-user research, HIV prevention, Product attributes, Africa, Microbicides, African women

## Abstract

**Introduction:**

Incorporating end-user input into the design of new vaginal microbicides for women is key to optimizing their uptake, consistent use, and, ultimately, success in combatting the heterosexual HIV epidemic.

**Methods:**

The Quatro Study assessed four placebo forms of vaginally inserted HIV-microbicides among young microbicide-naïve African women: on-demand film, insert and gel, and monthly ring. Participants randomly used each product for 1 month and provided product satisfaction ratings (1–5 scale), and opinions on product attributes and potential alternative designs. Qualitative data were collected through focus group discussions at study exit. Multivariable associations between attribute opinions and overall product rating were examined using Poisson regression models with robust standard errors to assess the attributes most influential to satisfaction.

**Results:**

Overall opinions of products and their individual attributes were generally positive; all products were rated either 4 or a 5 by ≥ 50% of participants. Attributes related to ease of use and interference with normal activities were the most salient predictors of satisfaction. Preferences for duration of use tended toward relatively shorter use periods for the ring (i.e., 1–3 months vs. 12 months) and for coitally independent dosing for the on-demand products.

**Conclusions:**

How well a product fit in with participants’ lifestyles was important to their overall satisfaction. For on-demand products, greater flexibility around timing of use was desired, to avoid coital dependency of the dosing.

**Electronic supplementary material:**

The online version of this article (10.1007/s10461-019-02576-8) contains supplementary material, which is available to authorized users.

## Introduction

Despite technological advances in HIV prevention approaches, the risk of HIV acquisition among young women in sub-Saharan Africa remains unacceptably high [1]. Over the past decade, several promising biomedical HIV prevention approaches, including vaginal microbicides and oral pills, have been evaluated in sub-Saharan Africa in phase III trials. However, several of these trials were unable to demonstrate effectiveness, or only showed limited levels of protection, in large part due to low product adherence among participants [2–8].

Microbicides applied inside the vagina in various delivery forms, including films, gels, suppositories or inserts, and rings, could provide women with a range of biomedical HIV prevention options [4, 8–11]. These formulations carry the benefits of being discreet, female-initiated, and could potentially be used without male partner consent. However, they must be liked and consistently used to optimize their impact. Adherence and acceptability have been conceptualized as distinct but closely linked [12]. An HIV prevention product that will impact the heterosexual epidemic in Africa must not only be biologically efficacious, but also suitable for the target population of “end-users” from a socio-behavioral perspective [13].

Therefore, focus has increased in HIV-prevention research toward gathering and incorporating end-user input to improve product designs. A key goal of end-user research during product development is to identify modifiable features, product attribute-related challenges, and barriers to product use, to ideally address these issues prior to efficacy trials. Improved HIV prevention product design and understanding of the context of product use among populations at high risk of HIV should improve the likelihood of adherence in trials and maximize the product’s uptake after licensure [10].

The Quatro Study assessed acceptability and use of four placebo vaginal delivery forms: film, insert (i.e., vaginal tablet), gel, and ring among young women in Southern Africa. This paper explores several dimensions of product acceptability, including ratings of how much each of the Quatro products was liked and the attributes of products that were acceptable or not. These analyses aim to shed light on the attributes that were salient drivers of overall product satisfaction, and to explore changes that might make vaginal delivery forms more acceptable to women in the future.

## Methods

### Study Design and Sample

The Quatro Study clinical component was a two-stage randomized cross-over acceptability study. In stage one, participants used each of four placebo products for 1 month and returned to the clinic monthly to provide their opinions. Stage two involved choosing one of the four products to use with every sex act for one additional month, with the goal of examining adherence. Here, we present findings from stage one only, which was designed to gather detailed feedback on participants’ experiences and satisfaction using each product.

Participants were recruited in Durban, South Africa, from a family planning clinic in the city center and the surrounding community; and in Chitungwiza, Zimbabwe, from family planning clinics, voluntary counseling and testing (VCT) clinics, and community locations such as markets, shopping centers, and informal settlements. One hundred participants were enrolled in each country (total N = 200) between June and November 2016.

Eligibility criteria were designed to enroll young potential end-users of vaginal microbicide products. Participants were HIV-negative, sexually-active women between the ages of 18 and 30. Pregnant women and those wanting to become pregnant in the next 6 months were excluded, as were those who had previously participated in microbicide or pre-exposure prophylaxis (PrEP) research. Pregnancy and HIV status were confirmed through laboratory testing at screening.

All study procedures received ethics and regulatory approval in both countries prior to implementation, and all participants provided written informed consent before undergoing any study procedures. At every visit, participants received HIV-risk reduction counseling, were offered condoms, and received explanations reminding them that the products were placebos and provided no protection.

### Study Products

**Fig. 1:**
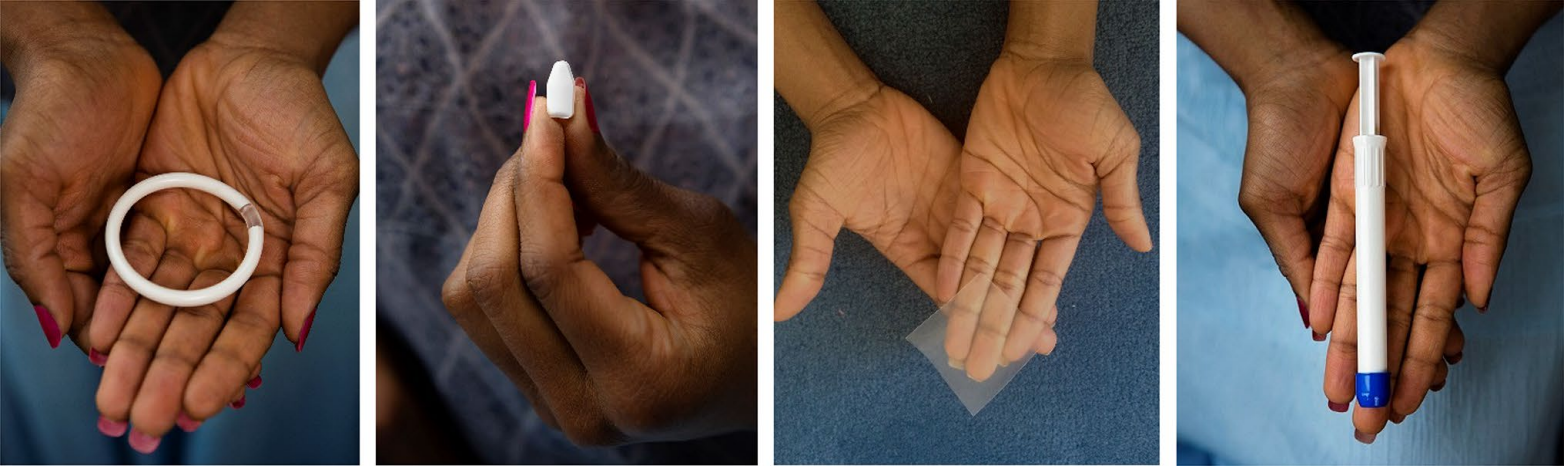
Quatro Study vaginal products: ring, insert, film, gel. Placebo vaginal delivery forms used in the Quatro Study: a polyurethane ring, a water-based vaginal insert or tablet, a dry gel film, and hydroxyethyl cellulose (HEC) gel (inside an applicator). Additional details on product size: The ring is 55.0 mm in diameter, with a cross-sectional diameter of 5.5 mm. The insert is approximately 7 mm × 15 mm. The film has dimensions of approximately 5 cm × 5 cm, with an approximate thickness of 100–120 µm. The gel applicator contains approximately 4 ml of gel

The delivery forms under study in Quatro were all inserted vaginally and included: an on-demand (i.e., used whenever required, in this case, precoitally) gel, insert, and film; and a monthly ring (Fig. 1). Products were provided by CONRAD in Arlington, Virginia, USA. Because the objective of Quatro was to explore delivery form acceptability, uncoupled from concerns about side effects or efficacy that may accompany an active drug, only placebo versions of the products were used.

### Procedures

After enrollment and a baseline questionnaire that collected demographics, behavioral, and product rating information prior to use, participants used all four products for 1 month each in a randomized sequence. In the months they were assigned to use one of the three on-demand products, women inserted the first dose in the clinic with guidance from the clinician, and then received four additional doses to use at home. They were instructed to insert the product in the vagina once a week up to 2 h before sex or insert the product without sex in any weeks where sex did not occur. The ring was inserted and removed with clinician guidance in the clinic, then inserted once more by the participant herself, with instructions to keep the ring inserted for the entire month. Participants returned to the clinic monthly to receive their next assigned product and complete interviewer-administered acceptability surveys about their experiences over the past month, which included product-specific questions, questions about opinions and preferences around HIV prevention products, and about recent sexual behavior.

A subset of participants in each country joined focus group discussions (FGDs) after their final month in the study. To ensure a variety of perspectives, 40 FGD participants were randomly selected after stratifying by product chosen in stage two. The FGDs were facilitated by a trained interviewer using a semi-structured guide. FGDs were audio-recorded and later transcribed into English.

Throughout the study, staff emphasized to participants the importance of honest feedback, and the impact of their role as product testers, co-designers, and research partners. During stage one, women were told that if they did not like using a product, they were not required to use it for the entire month. They were encouraged to communicate openly with the study staff about product experiences, specifically what they liked and disliked about each product.

### Measures

Demographic characteristics were collected at baseline with a standard questionnaire used in past microbicide and PrEP studies in Africa [2, 4]. A monthly acceptability questionnaire was developed based on formative research and input from the study team, including the product developer. After using each product for 1 month, women were asked for their opinions about its attributes, defined as product-specific characteristics, such as: physical appearance; timing or ease of use; feeling during insertion, sex; and menses. In addition, questions about potential modifications to the product were explored to assess whether a hypothetical alternative design would improve product satisfaction. Opinions about attributes and alternative product designs were provided on a 4-point scale from 1 (very unacceptable) to 4 (very acceptable). The primary outcome of this analysis was the participant’s overall satisfaction rating, defined as her assessment of how much she liked each product, rated on a 5-point Likert scale of 1 (dislike very much) to 5 (like very much). See the electronic supplement for sample questions from the monthly acceptability questionnaire.

The FGD guide covered questions around use experiences, product features that were most and least liked, and messaging for future marketing.

### Analysis

Participants who rated at least one product were included in the analysis. Demographic characteristics were tabulated by country and overall, and Pearson’s Chi square tests were used to assess differences by country.

The distributions of satisfaction ratings after 1 month of use were first examined and presented descriptively for each product. For multivariable analyses, the outcome of product satisfaction was dichotomized into rating the product as a 4 or 5 (“liking” the product) versus rating it as a 3 or lower (“not liking”). The distributions of opinions for nearly all attributes were generally skewed toward positive responses. Therefore, attribute opinions were dichotomized in descriptive and multivariable analyses as a 4 (“very acceptable”) versus 3 or lower (“not very acceptable”) The assessment of alternative product designs did not exhibit the same positive skew as the attribute opinions. As such, these responses were dichotomized into 3 or 4 (‘acceptable’, collapsing “very” and “somewhat” categories) versus 1 or 2 (‘unacceptable’, collapsing “very” and “somewhat” categories), to examine whether a proposed change was viewed negatively or positively overall.

A mixed-effect logistic regression model was used to predict the probability of liking each product by country; the model included a random intercept for randomization sequence and for subject to account for the clustered longitudinal structure of the data. To explore the relationship between product satisfaction and attribute opinions, relative risk ratios were modeled for each product using multivariable Poisson regressions with robust standard errors, controlling for country and month of product use. Age was also considered as a potential confounder. Variance inflation factors were used to assess the appropriateness of including all attributes in a single model for each product. All analyses were conducting using Stata [14].

For the qualitative analysis, a codebook was developed based on previous studies [15, 16] and an HIV-prevention product acceptability conceptual model [12]. Two analysts coded transcripts using Dedoose [17], a web-based qualitative analysis software. The analysts used regular inter-rater reliability tests to identify any discrepancies in coding, and all discrepancies were resolved by consensus. Based on the key findings from the quantitative analysis, reports incorporating codes corresponding to product attributes: “physical attributes,” “dosing regimen,” “route of administration,” “use attributes,” and “effect on life” were reviewed to further explore participants’ opinions and experiences regarding the Quatro products.

## Results

### Description of Study Sample

Of the 200 participants enrolled in the Quatro clinical study, 190 participants (95%) rated at least one product and are included in this analysis; 180 participants (90%) rated all four products. There were 40 women (21%) who participated in focus groups at the end of the study. Demographic characteristics of the analysis sample (N = 190) are presented by country in Table 1. The median age was 24 (interquartile range 21–26). Nearly all (93%) participants in South Africa and all (100%) in Zimbabwe had a primary partner at the time of study enrolment; although, only 11% of South African participants lived with, or were married to, their partners, compared with 94% in Zimbabwe (p < 0.001). Most (87%) participants had given birth at least once, and the majority (69%) had attained a secondary school education or higher. The samples in each country differed from each other on many baseline characteristics assessed, including experience with contraceptive methods.

**Table 1:** Baseline characteristics of participants who used all four vaginal placebo products in the Quatro Study, by country (N = 190)

Characteristic	South Africa (N = 91)	Zimbabwe (N = 99)	Total (N = 190)
	%	%	%
Age			
Median (IQR)—years	22 (20–25)	24 (22–26)	24 (21–26)
18–24	68	55	61
25–30	32	46	39
Currently has a primary partner*	93	100	97
Lives with partner or married***	11	94	54
Currently has a casual sex partner	9	2	5
Ever exchanged sex for money, goods, a place to stay, or services	3	2	3
Parity > 0***	73	100	87
Completed secondary school	76	63	69
Earns an income***	17	52	35
Attends religious services at least once a week***	68	100	85
No food insecurity in the past 4 weeks***	52	86	70
Has place for privacy in home	92	96	94
Worried she will contract HIV in next 12 months			
Not at all/a little	51	47	48
Somewhat/very/extremely	50	54	52
Contraceptive methods ever used			
Male condom***	92	60	75
Female condom*	1	7	4
Pills***	31	87	60
IUD	3	2	3
Implants***	15	49	33
Injectable***	70	33	51

*p < 0.05; **p < 0.01; ***p < 0.001 in Chi squared test

### Distributions of Product Satisfaction Ratings

**Fig. 2:**
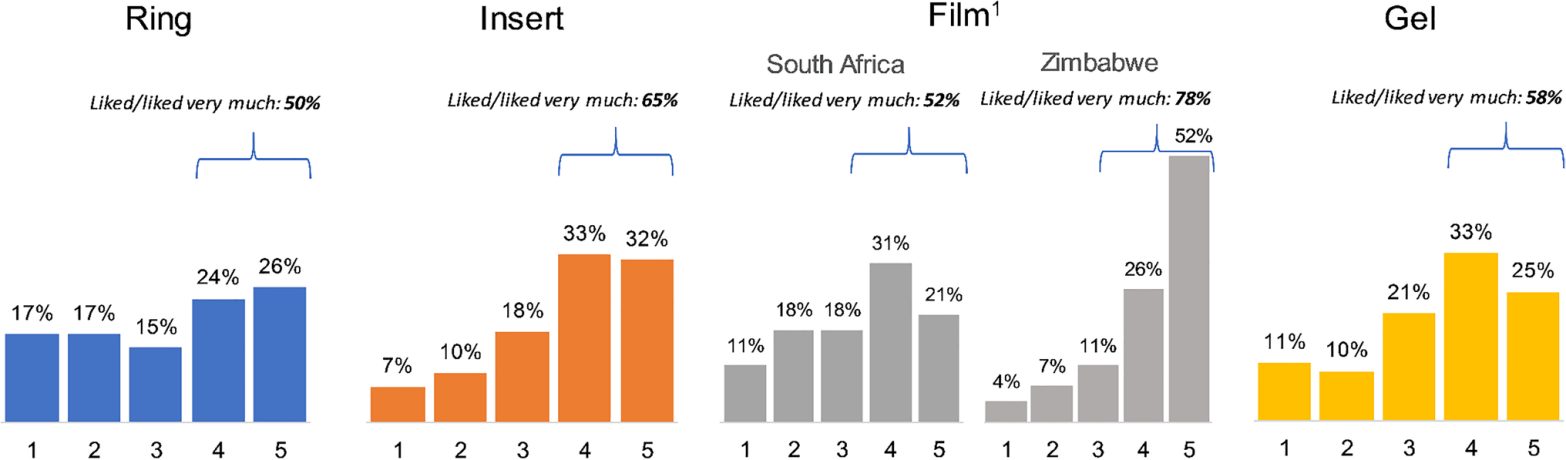
Distributions of Quatro product satisfaction ratings after 1 month of use (1 = disliked very much; 5 = liked very much). ^1^The distribution of ratings for the film differed significantly by country (p < 0.001)

The distributions of ratings for each product after 1 month of use are presented in Fig. 2. Overall, the products were well liked: ≥ 50% of participants rated each product either a 4 or 5. Of the 180 who rated all products, all but 2 (99%) rated at least one product 4 or 5, but there was variability in which products were liked. Only 12% rated all products 4 or higher.

The probability of liking the film differed significantly by country, with 78% of women in Zimbabwe assigning it a 4 or 5, compared to 52% in South Africa (p < 0.001). Product satisfaction ratings for the other three products did not differ significantly by country. Overall, the film and insert were rated most favorably, with 65% saying they liked each of the two products. The ring received the fewest favorable ratings; 50% liked the product (p = 0.001).

Detailed results from stage two, when women were asked to choose a product to use for the final month of the study, are presented elsewhere [18]. In brief, 29% chose the film, 28% chose the ring, 26% chose the insert, and 16% chose the gel; Zimbabwean women were significantly more likely to choose the film (45% vs. 13% in South Africa). Nearly all (91%) chose a product that they liked (rated 4 or 5 in stage one).

### Product Attribute Opinions

Table 2 presents the proportion of participants who said each attribute was “very acceptable,” by product. In general, women reported most of the product attributes as very acceptable, with some notable exceptions: less than half said the way the film felt in their hands (49%), the gel’s appearance (45%), and the timing of the insert (49%) and gel (45%) were very acceptable. Of note, shortly after the study launched, study staff reported that some women were misunderstanding the instruction to use the insert, film, and gel “up to 2 h before sex”, particularly in South Africa: some participants believed they needed to wait for 2 h after inserting the product before they could have sex. The instructions were clarified soon after the issue was uncovered, but this misperception may have persisted and may have impacted some women’s opinions on the timing.

**Table 2:** Proportion of Quatro participants who reported each product attribute (characteristic) as “very acceptable^a^,” by product

Product attribute	Ring	Insert	Film	Gel
	(N = 185)^b^	(N = 184)	(N = 186)	(N = 186)
	N (%)	N (%)	N (%)	N (%)
Aspects of use experience				
Interference with normal activities	113 (61)	146 (79)	146 (78)	138 (74)
Ease of use	99 (54)	144 (78)	112 (60)	151 (81)
Ease of storage	111 (60)	153 (83)	150 (81)	150 (81)
Timing of use^c^	101 (55)	90 (49)	97 (52)	84 (45)
Physical features				
Appearance	54 (29)	109 (59)	99 (53)	84 (45)
Size	69 (37)	155 (84)	104 (56)	115 (62)
Aspects of feeling and sensation				
Reported using during menses	117 (63)	69 (38)	56 (30)	66 (35)
Feeling during menses^d^	78 (67)	48 (70)	34 (61)	38 (58)
Reported using during sex	155 (84)	154 (84)	153 (82)	155 (83)
Feeling to participant during sex^d^	95 (62)	115 (75)	114 (75)	88 (57)
Reported using during sex and disclosed to partner	129 (70)	121 (66)	112 (60)	122 (66)
Feeling to partner during sex^d^	80 (62)	92 (76)	85 (76)	73 (60)
Feeling in hands	65 (35)	123 (67)	91 (49)	94 (51)
Feeling of insertion into vagina	89 (48)	134 (73)	94 (51)	100 (54)

^a^Possible response options were “very unacceptable,” “somewhat unacceptable,” “somewhat acceptable,” or “very acceptable”

^b^The sample size of women completing the acceptability questionnaire differs slightly for each product due to loss to follow up

^c^Timing of use was presented as the following for ring: “leaving the ring in for an entire month”; and for insert/film/gel: “having to use the [product] up to 2 h before sex”

^d^Of participants who reported use during menses/sex

Fewer women found specific attributes of the ring “very acceptable”. For example, only the following proportions of women found the appearance (29%), feeling in hands (35%), size (37%), and feeling inserting into the vagina (48%) as very acceptable.

### Relationship Between Attribute Acceptability and Overall Product Satisfaction

Multivariable models were constructed to examine the association between overall satisfaction with the product and acceptability of product attributes (Table 3). The likelihood of rating an attribute “very acceptable” did not differ by age as a continuous variable or by age group (18–24 vs. 25–30, all p > 0.25), so age was not included in the final models. Variance inflation factors did not indicate any significant correlation between attribute opinions per product (data not shown), hence all attributes were included in the models. Attribute associations, grouped by category, are further explained below. Illustrative quotes from qualitative FGD were used to provide examples of participants’ feedback about the attributes, in their own words.

**Table 3:** Multivariable associations between reporting individual product attributes “very acceptable^a^” and overall satisfaction with the product^b^

Product attribute	Model 1: ring N = 185^c^		Model 2: insert N = 182		Model 3: film N = 186		Model 4: gel N = 185	
	RR	(95% CI)	RR	(95% CI)	RR	(95% CI)	RR	(95% CI)
Aspects of use experience								
Interference with normal activities	2.14**	(1.33–3.43)	2.83**	(1.44–5.58)	1.96**	(1.20–3.21)	2.11**	(1.26–3.56)
Ease of use	1.58*	(1.07–2.34)	2.39**	(1.33–4.29)	1.56**	(1.16–2.09)	1.42	(0.82–2.48)
Ease of storage	1.25	(0.85–1.83)	0.94	(0.66–1.33)	0.94	(0.66–1.33)	0.85	(0.59–1.23)
Timing of use	2.24**	(1.42–3.51)	1.16	(0.93–1.45)	0.91	(0.67–1.24)	1.00	(0.69–1.44)
Physical features								
Appearance	1.17	(0.83–1.65)	1.13	(0.85–1.50)	1.23	(0.93–1.63)	1.52*	(1.09–2.10)
Size	0.92	(0.66–1.28)	1.17	(0.83–1.64)	1.05	(0.79–1.40)	0.83	(0.65–1.07)
Aspects of feeling and sensation								
Feeling during menses^d^	1.13	(0.77–1.65)	1.10	(0.74–1.63)	2.00*	(1.12–3.56)	0.88	(0.61––1.28)
Feeling to participant during sex^e^	0.90	(0.58–1.41)	1.23	(0.82–1.84)	1.39	(0.90–2.16)	1.10	(0.74–1.64)
Feeling to partner during sex^f^	1.09	(0.70–1.68)	1.45	(0.81–2.62)	0.94	(0.68–1.29)	1.63*	(1.03–2.57)
Feeling in hands	1.08	(0.70–1.65)	0.88	(0.65–1.19)	1.14	(0.92–1.43)	0.97	(0.73–1.28)
Feeling of insertion into vagina	1.14	(0.75–1.74)	1.00	(0.69–1.43)	1.00	(0.78–1.29)	1.18	(0.86–1.60)

*p < 0.05; **p < 0.01

^a^Versus reporting it was very/somewhat unacceptable or somewhat acceptable

^b^Liking the product was defined as rating it a 4 or 5 on a 5-point scale

^c^The sample size of women completing the acceptability questionnaire and being included in multivariate analyses differs slightly for each product due to loss to follow up and missing data

^d^Reference group includes women who did not report using the product during menses; this group did not rate the product differently from those who used with menses

^e^Reference group includes women who did not report using the product with sex; this group did not rate the product differently from those who used with sex

^f^Reference group includes women who did not report using the product during or did not disclose to their partner, this group did not rate the product differently from those who used with sex and disclosed use to their partners

#### Aspects of Use Experience

Across all four products, low perceived interference with normal activities was a statistically significant predictor of positive product rating (Table 3; p < 0.01 for all products). The salience of this association was echoed in the qualitative data in both countries. For example, in one FGD, a participant said of the ring, “And once you insert it, it will not affect anything. You will just do your work as usual and you will not feel it even the movements.” (Zimbabwe, age 24) This sentiment about the ring was echoed by another participant: “People will like it because you don’t have to keep inserting, inserting and inserting it.” (South Africa, age 24)

Ease of storage was not significantly associated with product rating (Table 3; p > 0.40 for all products). Reporting that the product’s ease of use was ‘very acceptable’ was significantly associated with liking the ring (RR 1.58, 95% CI 1.06, 7.51; p = 0.02), insert (RR 2.39, 95% CI 1.33, 4.29; p < 0.01), and film (RR 1.56, 95% CI 1.16, 2.09; p < 0.01); the association was similar for the gel although not statistically significant (RR 1.42, 95% CI 0.82, 2.48; p = 0.21). In describing their experiences with product use, women spoke about differing characteristics (depending on the product) that facilitated ease of use. Women who were happy with the ring pointed to its benefit of only needing to be inserted once at the beginning of the month. One participant said, “It is easy to use because you insert it once. You don’t have to worry about a next dose.” (South Africa, age 21) Another favored the film, “Because it is invisible, and you are even able to hide it. And it is easy to use it.” (South Africa, age 23) Another participant liked the insert’s discreetness and ease of use:

…Knowing that you are going to meet [have sex] with your partner, you could just put it in your bra. It is something portable, not difficult, you would just slip it in. It was easy to insert and it was not difficult to dissolve. (Zimbabwe, age 25)

Some women found the gel easy to use due to the applicator, and one woman in South Africa discussed being “used to… vaginal applicators given at the clinic” (South Africa, age 30), such as for vaginal yeast treatments.

Opinions about timing of use were not associated with overall rating for the three on-demand products, but women who found leaving the ring in for an entire month very acceptable were significantly more likely to like the ring (RR 2.24, 95% CI 1.42, 3.51, p < 0.01). One woman expressed liking this aspect of the ring, saying it was

good because you would just wear it once the whole month. No one would even imagine what will be going on. You won’t be seen poking fingers in the vagina, or to say I have forgotten or I have done this. (Zimbabwe, age 29)

#### Physical Features

Physical appearance was significantly associated with the gel rating (RR 1.52, 95% CI 1.09, 2.10, p = 0.01), but not with rating of the other three products. One FGD participant described concerns she had about the gel based on the appearance of the applicator, saying, “The shape of it I thought it would hurt me when inserting it. And how am I going to push this thing [the applicator].” (South Africa, age 26). In contrast, women discussed liking that the insert was a familiar-looking product (like oral pills), which made it look less “scary.”

In FGDs, women who disliked the ring discussed experiencing fear as well as difficulty and discomfort with insertion due to its size. However, opinion of product size was not a significant predictor of the overall product rating (Table 3; p > 0.15 for all products).

#### Aspects of Feeling and Sensation

High acceptance of how the film felt during menses was significantly associated with its overall rating (RR 2.00, 95% CI 1.12, 3.56; p = 0.02), but this relationship did not emerge with the other three products. Discussions of how the products felt during menses were not explored and did not arise spontaneously in the FGDs. Of note, only around one-third of participants (30–38%) reported using the on-demand products during menses.

Although quantitative opinions of product insertion feelings were generally not significant predictors of overall product rating, discourse in the focus groups revealed strong feelings and preferences; the physical feeling of the products during use and their dissolving properties were discussed extensively. Women who disliked the ring pointed to problems with feeling the ring slipping out, causing pain, itching, bleeding, or discomfort during daily activities. Others described liking the insert because it dissolved quickly. When discussing the film, participants cited problems with the film dissolving, stickiness, and the edges and corners of the film “poking” or “pricking” during insertion. Other FGD participants complained about the gel feeling cold and causing a lot of discharge that takes some time to come out.

The product attribute of feeling during sex was more salient in the qualitative discussions than in the quantitative analyses, which did not reveal any significant associations with product rating (p > 0.18 for all products). In the FGDs, women spoke about use of all four products during sex, with broad discussions of feeling that product use resulted in changes to the vaginal state (e.g. wetness, dryness, or tightness). However, there were no consistent trends in certain products causing particular vaginal changes, nor was there an overall pattern regarding whether these changes were seen as negative or positive. Some FGD participants said the ring increased sexual pleasure for themselves and/or their partners, while others cited discomfort during sex as a reason for not liking the ring. Women in both countries reported increased wetness due to the insert, which was a positive feature for some women, improving sex, and a negative for others. In contrast, others experienced the insert dissolving slowly, and feeling “sandy” during sex. Some women discussed the film causing dryness during sex or sticking to their partner’s penis after sex. Some complained about tightness during sex due to the film, although most saw this as a benefit, reporting overall positive experiences with film use during sex. As one woman explained, *“*the film is good because it shrinks you back to your virginity.” (Zimbabwe, age 26) Some women explained their preference for the gel due to the increased lubrication it provided during sex.

Finally, participants who said the way the gel felt during sex to their partner was very acceptable were significantly more likely to like it overall (RR 1.63, 95% CI 1.03, 2.57; p = 0.04). In the FGDs, women described the gel being easily felt by their partner due to the increased wetness it caused. For some, this was a benefit, increasing pleasure during sex, while for others, this caused concerns, such as for this participant: “For me gel was easy on application, but it was not as good on sex because my husband would say it feels like you already had sex, he did not like that slipperiness.” (Zimbabwe, age 25)

### Alternative Product Designs

Opinions of alternative product designs are presented in Table 4. In general, a majority of women felt the proposed alternatives would be acceptable. However, there were a few less popular alternatives.

**Table 4:** Opinions of alternative product designs after 1 month of use

How acceptable would it be to you to have a (product)…	Somewhat/very acceptable
	N (%)
Ring	(N = 185)^a^
…that changes to show how much of the drug has been released over time?	162 (88)
…that you could leave in for 3 months instead of 1 month?	114 (62)
…that you could leave in for 12 months instead of 1 month?	84 (45)
Insert	(N = 184)
…with a color other than white?	84 (46)
…that you use up to 1 day before sex instead of up to 2 h before sex?	160 (87)
…that you use up to 1 week before sex instead of up to 2 h before sex?	154 (84)
…that you use after sex instead of up to 2 h before sex?	115 (63)
Film	(N = 186)
…that is a color other than clear?	68 (36)
…that you use up to 1 day before sex instead of up to 2 h before sex?	164 (88)
…that you use up to 1 week before sex instead of up to 2 h before sex?	148 (79)
…that you use after sex instead of up to 2 h before sex?	118 (63)
Gel	(N = 186)
…that is thicker?	80 (43)
…that you use up to 1 day before sex instead of up to 2 h before sex?	163 (88)
…that you use up to 1 week before sex instead of up to 2 h before sex?	147 (79)
…that you use after sex instead of up to 2 h before sex?	127 (68)

^a^The sample size of women completing the acceptability questionnaire differs slightly for each product due to loss to follow up

Women preferred the color of the insert and film they used in the study to the proposed alternatives, with less than half (46%) saying an insert with a color other than white would be acceptable, and only 36% approving of a film that is not clear. Less than half (43%) felt a gel with a thicker consistency would be acceptable. One suggestion for improving the film that emerged from the FGDs was to make it round, rather than square, to eliminate corners that could poke the vagina. Some women in FGDs recommended offering rings of differing sizes to be “compatible” for different sized women.

Alternate timing requirements for the on-demand products were generally acceptable, with the highest proportion (87–88%) stating use up to 1 day before sex would be acceptable. For example, one FGD participant expressed this preference, saying, “Maybe it has passed [2 h is over], maybe it is almost 6 h and you are also irritated. You see, the thing which I have inserted is no longer effective. A day is just alright.” (South Africa, age 28) Overall, using an insert, film, or gel after sex was less favorable relative to other proposed alternatives, but still acceptable to approximately two-thirds of participants for each product (63–68%).

Interest in the ring declined with increased dosing duration, with 62% indicating a 3-month ring would be acceptable and 45% indicating a 12-month ring would be acceptable. FGD participants in both countries indicated they would accept a ring worn for more than 1 month if it were more comfortable—smaller in size, more flexible, and designed so that it doesn’t move around in the vagina. A participant explained:

Let it [the ring] be changed also and become a bit smaller or even the texture, can it be made into something slippery so that it becomes easy to insert [laughs], and even softer. Haa! that way even if it is to spend 3 months or the whole year inserted it will still be fine. (Zimbabwe, age 26)

Reacting to the proposed longer duration, one participant (South Africa, age 24) also mentioned concerns about STIs or smells that could require monthly changing of the ring.

## Discussion

In this study we uncovered three key findings about the acceptability of four placebo vaginal microbicides among young women in Southern Africa. First, all the products were generally well-liked, and opinions of individual product attributes were also positive. Secondly, attributes related to the ease of use and (non-)interference with normal activities were the most salient predictors of overall product rating. Finally, preferences for timing of use revealed two important points. First, longer periods of use are not always the most acceptable, as the 1- and 3-month rings were more favorable than the 12-month ring. Secondly, for single-use products, timing that would not require coital dosing emerged as the most favored regimen, in part due to the spontaneity of sex, but also driven by desires for the product to have sufficient time to fully dissolve or disappear and not be noticeable during sex. This feedback expressed by some participants in FGDs raises a potential concern regarding the feasibility of meeting this need for discretion with an effective microbicide, given that the active pharmaceutical ingredients must still be present in the vagina for them to work.

In previous studies assessing acceptability of potential intravaginal methods for HIV prevention, including diaphragms, gels, and rings, women’s opinions have generally been very positive, particularly when measured quantitatively [8, 19–22]. Here, we found that women reported liking all the products, with the film more highly rated in Zimbabwe. In addition, though satisfaction was generally high, it was more nuanced than previously reported in studies of similar products. We hypothesize this was due to our messaging to women focused on their role as co-designers and the importance of candid feedback, especially telling us what they did not like. Many women in this study were willing to report that they did not like a product, which increased our confidence in their candor. However, an alternative explanation may be that the single month of use (in contrast to longer periods in other studies) did not provide sufficient time for women to grow accustomed to the products. It is a possibility that ratings may have differed given longer periods of use. In general, individual attributes were also acceptable to our participants, though some specific features were less favorable, more so for the attributes of the ring than the other products.

An important aspect of end-user research to consider in light of these results is that positive product or attribute opinions do not guarantee consistent use of a product [23]. Other contextual factors within the conceptual realm of product acceptability must be considered to more fully understand women’s decision making processes involved in HIV prevention, and to ensure that beyond being liked, these products are optimized for correct and consistent use [12]. This disconnect has emerged in past studies; for example, two separate studies of diaphragms and gels in Southern Africa found high product acceptability but low consistent use, with factors related to male partners (refusal, disclosure, perceived approval) emerging as prominent drivers of use [22, 24]. In our multivariable models, acceptability of each product was associated with how easy and non-interfering their use was, suggesting a linkage between use and acceptability; male partners’ acceptability was only associated with ratings for the gel. Partner opinions emerged as a more influential factor in the focus group discussions. Of note, roughly 20% of women did not use the products with sex, which limited our ability to assess male partner influence.

Another key finding from these data was that use-related attributes, such as ease of use and interference with daily life, were more salient with product satisfaction than the physical features of the products. This result was likely due, in part, to the study design, in which participants were asked to use each product. Attributes relating to how well the product fit in with their lifestyle then became more evident. Physical features may play an important role during the “first impression” stage of product introduction, prior to uptake and usage. In a separate analysis from this study of product choice and preference [18], participant’s most preferred product shifted prior to and following introductory informational sessions and again after use, implying that information and experience changed opinions about the products. Our results corroborate other studies of female-controlled HIV prevention methods that have reported practical aspects of use to be an important driver of product acceptability and adherence [25, 26].

Participants had varied opinions about product timing. For on-demand products, greater flexibility was desired, mainly so that women could be prepared for situations when sex was not planned or scheduled. Participants most preferred an insert, film, or gel that could be used up to 1 day or 1 week before sex, to avoid the coital dependency of the dosing. This is consistent with previous studies that have highlighted challenges presented by women’s uncertainty around timing of sex and need for discretion [2, 27]. While 62% of participants said a ring that would stay inserted for 3 months as opposed to 1 month would be acceptable, a higher proportion accepted the timing of the 1-month ring. When longer durations of use (1 year) were proposed for the ring, the proportion stating the duration would be acceptable decreased. While there is some evidence that longer-acting HIV prevention products, such as injections, are more favored by end-users [28–32], longer may not translate into better for the ring, as women may not want to leave the ring inserted beyond 1 month due to hygiene or comfort concerns. This nuance warrants closer examination in future research to determine the optimal duration for different products, as well as counseling around hygiene, acceptable intermittent removals for menses and other concerns, given that longer-acting products are currently in development [33].

## Strengths and Limitations

A major strength of this study is that we collected female participants’ opinions of the four delivery forms following actual experiences with the products, rather than hypothetical product presentations. We recruited sexually active women only, and instructed them to use the products with sex, which allowed us to gather feedback based on the full product use experience and explore the potential influences of male partners’ perspectives. In addition, because this study was not a clinical trial, we did not strongly emphasize adherence to the dosing regimens, which possibly enhanced participants’ openness and honesty to provide product feedback. Less pressure to perfectly adhere to product use may have led to less misreporting of adherence or inflated acceptability measures reported in clinical microbicide trials [34]. As described above, throughout the study, staff reiterated the participants’ role as co-designers and reminded them that their feedback about not only what they liked, but what they did not like, could inform the product design for other women in the community.

Nevertheless, this study has several limitations. Given the sample size, statistical power was insufficient to explore nuances around product attributes that were well-liked overall. Although the use of placebo products was by design to focus opinions on the delivery form separated from the active pharmaceutical ingredients, this nevertheless limits the generalizability of our results to eventual active products. Our design prevents us from evaluating acceptability and tolerability of side effects that may accompany active products, or, conversely, the reward of potential or certain protection from HIV. As mentioned above, not all participants used the products during sex, limiting our ability to assess the products the way they would be used in a real world setting. Finally, the brief period of misunderstanding surrounding how to use the precoital products at the beginning of the study may have impacted some participants’ opinions of the on-demand products. Nevertheless, the on-demand products were rated highly.

## Conclusion

A key message from this end-user study is that various intravaginal dosage forms of HIV prevention, administered both on-demand and monthly were liked, with no clear favorite. The study highlighted the importance of offering vaginal microbicide products that can easily be used and incorporated into women’s lifestyles. Ideally, like contraceptives, an array of on-demand and longer-acting HIV prevention products will be available to appropriately cater to the varied needs of women throughout different stages of their sexual relationships and reproductive lives.

## Electronic supplementary material

Below is the link to the electronic supplementary material.

Supplementary material 1 (DOCX 13 kb)
