# Supplementary material for: Perspectives from Young South African and Zimbabwean Women on Attributes of Four (Placebo) Vaginal Microbicide Delivery Forms
Source: AIDS Behav. 2019 Jun 28;24(2):637–47. doi: 10.1007/s10461-019-02576-8 (PMC6988116; doi:10.1007/s10461-019-02576-8)
Supplement: Supplementary file 1 — Supplementary material 1 (DOCX 13 kb) [file 10461_2019_2576_MOESM1_ESM.docx]

**Supplemental Table 1: Wording of questionnaire items selected for attribute acceptability analyses^1^**

| ***Product rating***  Please rate how much you liked the (PRODUCT) you used over the past month on a scale of 1-5. |
| --- |
| ***Aspects of use experience***  What is your opinion of how much using the (PRODUCT) interfered (got in the way of or caused problems) with your normal activities during the past month?  What is your opinion of the ease of using the (PRODUCT)?  What is your opinion of the ease of storage of the (PRODUCT)?  What is your opinion of leaving the ring in for an entire month?  What is your opinion of having to use the insert/film/gel up to two hours before sex? |
| ***Physical features***  What is your opinion of the way the (PRODUCT) looked?  What is your opinion of the size of the ring/insert/film/gel applicator? |
| ***Aspects of feeling and sensation***  What is your opinion of how the (PRODUCT) felt during sex to you?  What is your opinion of how the (PRODUCT) felt during menses?  What is your opinion of how the (PRODUCT) felt during sex to your partner?  What is your opinion of how the (PRODUCT) felt in your hands?  What is your opinion of how it felt to put the (PRODUCT) into your vagina? |

^1^Questionnaires were translated into the local language (Shona in Zimbabwe and isiZulu in South Africa) and administered by a trained interviewer who read each question aloud, substituting the product relevant to the visit month where “(PRODUCT)” is denoted.
